# Network expansion of genetic associations defines a pleiotropy map of human cell biology

**DOI:** 10.1038/s41588-023-01327-9

**Published:** 2023-02-23

**Authors:** Inigo Barrio-Hernandez, Jeremy Schwartzentruber, Anjali Shrivastava, Noemi del-Toro, Asier Gonzalez, Qian Zhang, Edward Mountjoy, Daniel Suveges, David Ochoa, Maya Ghoussaini, Glyn Bradley, Henning Hermjakob, Sandra Orchard, Ian Dunham, Carl A. Anderson, Pablo Porras, Pedro Beltrao

**Affiliations:** 1 European Molecular Biology Laboratory, European Bioinformatics Institute (EMBL-EBI), Cambridge, UK; 2 Open Targets, Cambridge, UK; 3 Wellcome Sanger Institute, Cambridge, UK; 4 Computational Biology, Genomic Sciences, GSK, Stevenage, UK; 5 Institute of Molecular Systems Biology, ETH Zürich, Zürich, Switzerland

**Keywords:** Genome-wide association studies, Data mining

## Abstract

Interacting proteins tend to have similar functions, influencing the same organismal traits. Interaction networks can be used to expand the list of candidate trait-associated genes from genome-wide association studies. Here, we performed network-based expansion of trait-associated genes for 1,002 human traits showing that this recovers known disease genes or drug targets. The similarity of network expansion scores identifies groups of traits likely to share an underlying genetic and biological process. We identified 73 pleiotropic gene modules linked to multiple traits, enriched in genes involved in processes such as protein ubiquitination and RNA processing. In contrast to gene deletion studies, pleiotropy as defined here captures specifically multicellular-related processes. We show examples of modules linked to human diseases enriched in genes with known pathogenic variants that can be used to map targets of approved drugs for repurposing. Finally, we illustrate the use of network expansion scores to study genes at inflammatory bowel disease genome-wide association study loci, and implicate inflammatory bowel disease-relevant genes with strong functional and genetic support.

## Abstract (web summary)

Network expansion of trait-associated genes for 1,002 human phenotypes identifies pleiotropic gene modules and biological processes associated with multiple traits, and potential opportunities for drug repurposing and development.

## Main

Proteins that interact tend to take part in the same cellular functions and be important for the same organismal traits^1,2^. Through a principle of guilt-by-association, it has been shown that molecular networks can be used to predict the function or disease relevance of human genes^3–5^. On the basis of this, protein interaction networks can augment genome-wide association studies (GWAS) by using GWAS-linked genes as seeds in a network to identify additional trait-associated genes^6–9^. It is well known that GWAS loci are enriched in genes encoding for approved drug targets^10,11^ and genes linked to a trait by network expansion are similarly enriched, even when excluding genes with direct genetic support^12^. This is an opportune time to revisit the application of network approaches to GWAS interpretation on the basis of recent large improvements in the human molecular networks available, single-nucleotide polymorphism (SNP) approaches to gene mapping and the extent of human traits/diseases mapped by GWAS. In particular, there have been substantial improvements in the identification of likely causal genes within GWAS loci using expression and protein quantitative trait loci analysis^13,14^, as well as integrative approaches based on machine learning^11^.

The genetic study of large numbers of diverse human traits also opens the door to the study of pleiotropy, which occurs when a single genetic change affects multiple traits. Studying pleiotropy can help in the drug discovery process by either increasing the number of potential indications for a drug or avoiding unwanted side effects. Large-scale investigations of the most pleiotropic cellular processes have relied primarily on gene deletion studies. For example, yeast gene deletion studies have revealed pleiotropic cellular processes that include endocytosis, stress response and protein folding, amino acid biosynthesis and global transcriptional regulation^15^. Identification of these highly pleiotropic cellular systems highlights core conserved processes and the complex interconnections within cell biology. Human GWAS data have been extensively used to quantify pleiotropy at the SNP level^16–18^ and although this has shed light on the degree of pleiotropy and the relationship between traits, it has not often led to identification of the molecular mechanisms that underlie their common genetic basis.

Here, we augmented GWAS data for 1,002 traits by network expansion with the purpose of studying pleiotropic cellular processes at the level of the human organism. This network expansion recovers known disease genes not associated by GWAS, identifies groups of traits under the influence of the same cellular processes and defines a pleiotropy map of human cell biology. Finally, we illustrate the use of network expansion scores to characterize inflammatory bowel disease (IBD) genes at GWAS loci, and implicate IBD-relevant genes with strong functional and genetic support.

## Results

### Systematic augmentation of GWAS with network propagation

**Fig. 1:**
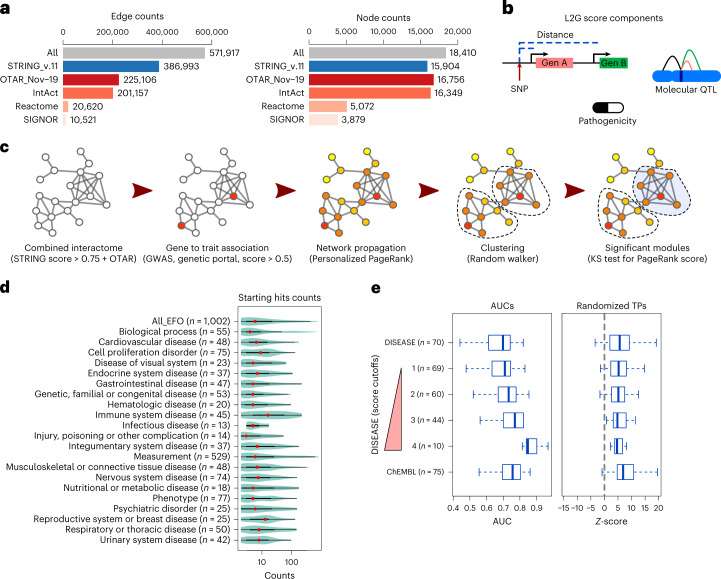
Implementation and benchmarking of network-based augmentation of GWAS. **a**, Edge and node counts of the combined interactome and its components. OTAR is the Open Targets combined physical protein interaction network that is provided via a Neo4j Graph Database. **b**, Graphic representation of some L2G components: SNP-to-gene distance, data from QTLs and variant effect predictions. The integration of information into the L2G score has been described previously^11^. **c**, Graphical representation of the network-based approach: network propagation of the initial input, clustering using a random walker to find gene communities and scoring of modules using the distribution of PageRank score. KS, Kolmogorov–Smirnov. **d**, Number of starting genes linked to traits, grouped in therapeutic areas. In the violin plot, the red dots represent the median, the limits of the thick line correspond to quartiles 1 and 3 (25% and 75% of the distribution) and the limits of the thin line are 1.5× the interquartile range. **e**, Benchmarking of the method, using as a starting signal genes from the Open Targets Genetics portal with a L2G score >0.5. AUC values are calculated using as positive hits the DISEASE database, with increasing cutoff values for its gene-to-trait score (Methods), as well as clinical trials data from the ChEMBL database (clinical phase II or higher). We also re-calculated the AUC values and determined *Z*-scores reflecting the deviation in AUCs relative to those observed after randomization of the list of true positives (TPs). In the boxplots, the middle lines represent the median, the limits of the box are quartiles 1 and 3 and the whiskers represent 1.5× the interquartile range.

Recent studies have shown that a comprehensive protein interaction network is critical for network propagation efforts^9^. Here, we combined the International Molecular Exchange physical protein interaction dataset^19^ from IntAct (protein–protein interactions)^20^, Reactome (pathways)^21^ and SIGNOR (directed signaling pathways)^22^. To facilitate re-use of these data (referred to as ‘OTAR interactome’) we have made the data available via a Neo4j Graph Database (ftp://ftp.ebi.ac.uk/pub/databases/intact/various/ot_graphdb/current). The physical interactions were combined with functional associations from the STRING database (v.11)^23^ to give a final network containing 571,917 edges connecting 18,410 proteins (nodes) (Fig. 1a). GWAS trait associations were mapped to genes using the locus-to-gene (L2G) score from Open Targets Genetics, a machine learning approach that integrates features such as SNP fine-mapping, gene distance and molecular quantitative trait locus (QTL) information to identify causal genes (Fig. 1b)^11^. Genes with L2G scores higher than 0.5 are expected to be causal for the respective trait association in 50% of cases.

For each GWAS, associated genes were used as seeds in the interaction network. Of 7,660 GWAS genes linked to at least one trait, 7,248 correspond to proteins present in the interaction network. We then used the Personalized PageRank (PPR) algorithm to score all other protein coding genes in the network where genes connected via short paths to GWAS genes receive higher scores (Fig. 1c). Genes in the top 25% of network propagation scores were used to identify gene modules, from which we selected those significantly enriched for high network propagation scores (Benjamini–Hochberg (BH)-adjusted *P* < 0.05 with Kolmogorov–Smirnov test) and with at least two GWAS-linked genes (Methods). We applied this approach to 1,002 traits (Supplementary Table 1) with GWAS in the Open Targets Genetics portal that had at least two genes mapped to the interactome. These GWAS were spread across 21 therapeutic areas, and differed in the number of GWAS-linked genes (median 6, range 2–763) (Fig. 1d).

To measure the capacity of the network expansion to recover trait-associated genes, we defined a ‘gold standard’ set of disease-associated genes (from https://diseases.jensenlab.org) that are known drug targets for specific human diseases (from the ChEMBL database, Methods). To avoid circularity in benchmarking, we excluded gold standard genes that overlapped with GWAS-linked genes for the respective diseases. The network propagation score predicted disease-associated genes with an average area under the receiver operating characteristic (ROC) curve (AUC) >0.7 for the most stringent definition of disease-associated genes as well as known drug targets (Fig. 1e and example ROC curves in Supplementary Fig. 1). The performance was higher than that observed with random permutation of the gold standard gene sets (Fig. 1e and Supplementary Fig. 2; true positive permutations), suggesting that it is not strongly biased by the placement of the gold standard genes within the network. We also tested the impact of changing the interaction network, either by using subsets of the network defined here or by using the previously defined composite PCNet network^9^ (Supplementary Fig. 3). Overall, the combined network performed best with an accuracy similar to that of the larger PCNet (Supplementary Fig. 3).

In total, we obtained network propagation scores for 1,002 traits and gene modules for 906 traits (Supplementary Table 1).

### Network propagation identifies related human traits

Identifying groups of traits likely to have a common genetic basis is of value because drugs used to treat one disease may also have effects in related diseases. Genetic sharing between human traits is often determined by correlation of SNP-level statistics from GWAS; however, this approach does not identify how the shared genetics corresponds to shared biological processes. In addition, many GWAS do not report the full summary statistics needed for such comparisons. By contrast, network propagation scores can be calculated from the set of candidate genes available for any GWAS. To benchmark trait–trait associations derived from network propagation, we used the similarity of annotations from the Experimental Factor Ontology (EFO), which include aspects of disease type, anatomy and cell type among others. For example, pairs of related neurological traits tend to share many annotation terms in the EFO. Using these annotations, we defined 796 pairs of traits that are functionally related and therefore likely to have a common genetic basis (Methods). An additional benchmark was obtained from trait-to-trait genetic correlations calculated from SNP-based analyses^24–26^. Using these benchmarks, we show that similarity in the network propagation scores can identify functionally and genetically related pairs of traits (Supplementary Fig. 4).

**Fig. 2:**
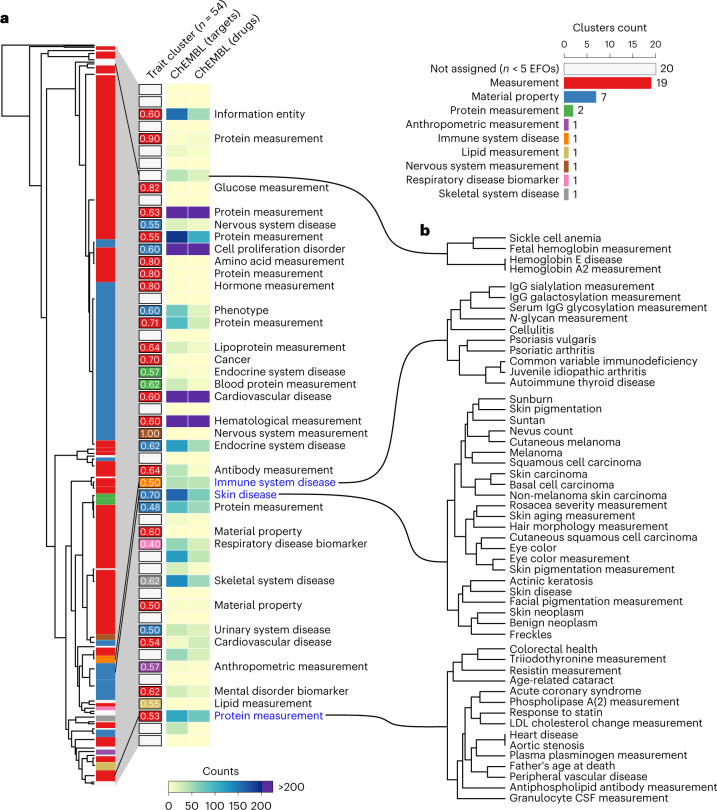
Trait–trait genetic and functional similarities determined from network expansion of GWAS data. **a**, Tree showing the Manhattan distance between all traits, using the full PPR score. Hierarchical clustering was performed using a cutoff of *h* = 1, leading to 54 clusters, colored depending on the predominant EFO ancestry term. The right-hand panel is a barplot showing the 54 clusters with the frequencies for the predominant EFO ancestry terms and a heatmap showing the counts for ChEMBL targets and drugs. The text label next to each cluster corresponds to the second most predominant EFO terms that, on average, label 35% of the traits within the clusters that have a text label. **b**, Examples of traits grouped using the Manhattan distance, extracted from the tree in **a**. CSF, colony-stimulating factor; Ig, immunolglobulin; LDL, low-density lipoprotein.

To explore trait–trait relationships on the basis of the similarity of their perturbed biological processes, we used the pairwise distance of network propagation scores to build a tree by hierarchical clustering (Fig. 2a), and defined 54 subgroups of traits. The traits tend to group according to functional similarity with 34 of 54 having an EFO term annotated to more than 50% of the traits in the group (Fig. 2a). In Fig. 2b we show examples of traits that are grouped together according to the network propagation scores. These include known relationships between immune-associated traits such as cellulitis or psoriasis and immunoglobulin G measurements; the relationship between skin neoplasms and skin pigmentation or eye color; or the clustering of cardiovascular diseases (acute coronary symptoms) with lipoprotein measurements and cholesterol.

We obtained drug indications from the ChEMBL database for the diseases in each cluster (Fig. 2a). This allows us to find clusters in which drugs may be considered for repurposing, as well as groups of traits in which drug development is most needed. Eighteen clusters representing 64 traits contain no associated drug and represent less well-explored areas of drug development. All trait clusters, genes and corresponding drugs are available in Supplementary Table 1.

### Pleiotropy of gene modules across human traits

**Fig. 3:**
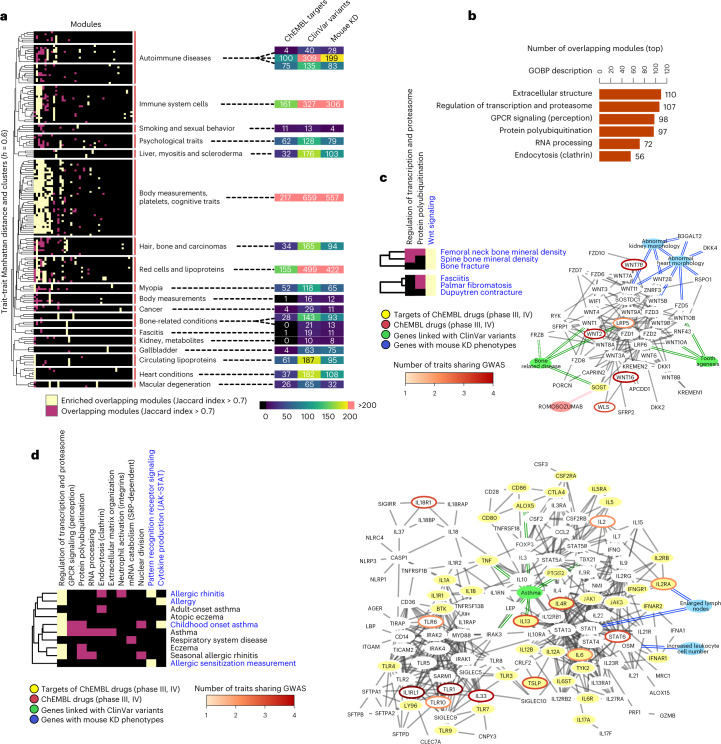
Multitrait gene module associations for studies of shared biological processes and drug-repurposing opportunities. **a**, Heatmap showing the overlap between gene modules across traits. Traits were clustered using hierarchical clustering (Methods) and subgroups were defined by a cutoff of 0.6 average correlation coefficient. A module was considered the same across different traits when most genes are in common (Jaccard index > 0.7). Significant trait–module relations are marked in yellow or pink, with yellow indicating modules overrepresented in one of the subgroups of traits (one-sided Fisher’s exact test, adjusted *P* < 0.05) and pink otherwise. The heatmap in the right-hand panel shows the number of genes in modules from each subgroup of traits that are drug targets (phase III or higher, ChEMBL database), linked with clinical variants (ClinVar database) or with mouse KO phenotypes (International Mouse Phenotyping Consortium database). **b**, Barplot showing the number of traits linked with the top six most pleiotropic gene modules. The GOBP description is based on the results of a GOBP enrichment test (Methods). **c**, Simplified heatmap of the clusters in **a** concerning bone-related and fasciitis traits. The represented network includes genes from the modules indicated in blue letters and the represented interactions have been filtered for visualization (Methods). Blue nodes, relevant mouse KO phenotypes; green nodes, diseases with clinical variants enriched in this gene module; red nodes, drugs in clinical trials. Genes linked to blue, green or yellow nodes have the linked mouse phenotypes, clinical variants in the linked disease or are targets of the linked drug. Genes that are the targets of drugs in clinical trials have yellow nodes. GWAS-linked genes (L2G score > 0.5) have borders colored in an orange to red gradient (count of GWAS-linked traits). **d**, Simplified heatmap of one the clusters in **a** concerning allergic reactions (node and edge color code are the same as in **c**). In this case, two modules were merged to build the interaction network in the right-hand panel. mRNA, messenger RNA; SRP, signal recognition particle.

We can study the pleiotropy of human cell biology by identifying which gene modules tend to be associated with many human traits. This allows us to understand how perturbations in specific aspects of cell biology may have broad consequences across multiple traits. In total, we found 2,021 associations between gene modules and traits, of which 886 (43.8%) are gene modules linked to a single trait and the remaining can be collapsed to 73 gene modules linked to two or more traits (Fig. 3a, Supplementary Table 2 and Methods). The 73 modules associated with more than one trait did not have a significantly larger number of genes (*P* = 0.72, Kolmogorov–Smirnov test), whereas the traits linked with the 73 pleiotropic gene modules tend to have a higher number of significant initial GWAS seed genes (Supplementary Fig. 5). Therefore, traits with a larger number of linked loci are more likely to be associated with pleiotropic gene modules.

The six most pleiotropic gene modules were linked to between 56 and 110 traits in our study, and were enriched (Gene Ontology Biological Process (GOBP) enrichment with one-sided Fisher’s exact test, BH-adjusted *P* < 0.05) for genes involved in protein ubiquitination, extracellular matrix organization, RNA processing and G protein-coupled receptor (GPCR) signaling (Fig. 3b). Gene deletion studies in yeast have identified some of the same cellular processes as being highly pleiotropic^15^. Genes within pleiotropic modules linked to ten or more traits are enriched in genes that are ubiquitously expressed (fold enrichment = 1.42, *P* = 1.71 × 10^−16^, Fisher’s exact test, one-sided), have many deletion phenotypes (fold enrichment = 1.56, *P* = 1.71 × 10^−30^, Fisher’s exact test, one-sided) and higher numbers of genetic interaction (Fisher’s exact test, one-sided *P* = 4.155 × 10^−^^10^). Targeting pleiotropic processes with drugs could, therefore, have broad application, but may also raise safety concerns. However, despite these enrichments, there is no simple correlation between the number of traits linked to a gene module and the enrichment of ubiquitously expressed genes (Pearson’s *r* = 0.0793) or genes with many deletion phenotypes (Pearson’s *r* = −0.0345). This analysis allows us to connect gene deletion phenotypes with human traits (Supplementary Fig. 6). For example, a pleiotropic module linked to traits such as ‘autism spectrum disorder’ and ‘osteoarthritis’ has a high fraction of gene deletion phenotypes impacting on protein transport, and a module linked with Alzheimer’s disease, balding measurement and bone density has genes with a high fraction of gene deletion phenotypes associated with cellular senescence (Supplementary Fig. 6).

We then related pleiotropy as defined by the module–trait associations derived here with pleiotropy defined by CRISPR gene deletion studies. For each Gene Ontology (GO) term, we calculated the enrichment in genes linked with many traits in our analysis with the enrichment in genes having many gene deletion phenotypes. GO terms specifically enriched in pleiotropic genes based on our definition are dominated by terms that relate to multicellularity, such as membrane signaling, cell-to-cell communication and cell migration (Supplementary Fig. 7). For pleiotropy that is specifically found with CRISPR screens, we find terms related to essential processes such as cell cycle, ribosome biogenesis and RNA metabolism (Supplementary Fig. 7).

For each of the 73 pleiotropic gene modules, we highlighted those that are overrepresented in each group of related traits (Fig. 3a and Methods, one-sided Fisher’s exact test, BH-adjusted *P* < 0.05). To facilitate the study of cell biology and drug-repurposing opportunities we annotated (Fig. 3a and Supplementary Table 2) the genes found in overlapping modules for each of the clusters with data from: ChEMBL (targets of drugs in at least phase III clinical trials), ClinVar (genes linked to clinical variants) and mouse knockout (KO) phenotypes (phenotypic relevance and possible biological link). We explore a few examples of these modules in the following sections.

### Shared mechanisms and drug-repurposing opportunities

We identified two groups of traits (bone and fasciitis related) that are predicted to have a common determining gene module (Fig. 3c and Supplementary Table 3). This module is enriched in Wnt signaling genes, which have been previously linked to bone homeostasis^27^ and to different types of fasciitis as well as Dupuytren’s contracture^28^. We collected genes harboring likely pathogenic variants from ClinVar (Methods), hereafter referred to as ClinVar variants. This gene module is enriched in genes harboring ClinVar variants from patients with tooth agenesis and bone-related diseases (osteoporosis and osteopenia). Several genes with ClinVar variants, such as *LRP6*, *SOST*, *WNT1*, *WNT10A* and *WNT10B*, are not linked to bone diseases via GWAS. Genetic manipulation of several genes within this module causes changes in bone density in mouse models^29^. In addition, this module contains the target (SOST) of Romosozumab, a drug proven effective to treat osteoporosis.

In a second example (Fig. 3d and Supplementary Table 3), we identified a group of ten respiratory (for example, asthma) and cutaneous (for example, eczema) immune-related diseases that share three gene modules: a highly pleiotropic module related to regulation of transcription and proteasome, and two more specific modules related to pattern recognition receptor signaling and cytokine production with Janus kinase/signal transducer and activator of transcription (JAK–STAT) involvement. These modules were significantly enriched (one-sided Fisher’s exact test, *P* < 0.05) in genes having likely pathogenic variants from patients with asthma. The two most specific gene modules were grouped and are shown in Fig. 3d highlighting several genes with known pathogenic variants not associated with these diseases via GWAS (for example, *IRAK3*, *TNF*, *ALOX5*, *TBX21*). *IRAK3*, encoding a protein pseudokinase, is an example of a druggable gene not identified by GWAS for asthma, but with protein missense variants linked to this disease^30^, and mice model studies have implicated the regulation of IRAK3 in airway inflammation induced by interleukin-33 (IL-33)^31^. Although no drug for IRAK3 is used in the clinic, this analysis suggests it may serve as a relevant drug target for asthma and other related diseases.

We identified a total of 41 targets of 126 drugs targeting the genes in the module shown in Fig. 3d. To identify drugs that could have repurposing potential, we excluded those already targeting therapeutic areas that include the ten diseases linked to this gene module. This resulted in 18 drugs (Supplementary Table 3) targeting 5 genes including: 14 drugs targeting *PTGS2*, used to treat primarily rheumatic disease and osteoarthritis; interferon alfacon1 or alfa-2B (targeting *IFNAR1* and *IFNAR2*), designed to counteract viral infections; galiximab and antibody for *CD80* (phase III trials for lymphoma); and the antibody RA-18C3 targeting IL1A for colorectal cancer. These drugs may be suited to repurposing for respiratory or cutaneous autoimmune-related diseases. As an example, RA-18C3 has shown benefit in a small phase II trial for hidradenitis suppurativa (acne inversa)^32^.

### Gene module analysis of related immune-mediated diseases

**Fig. 4:**
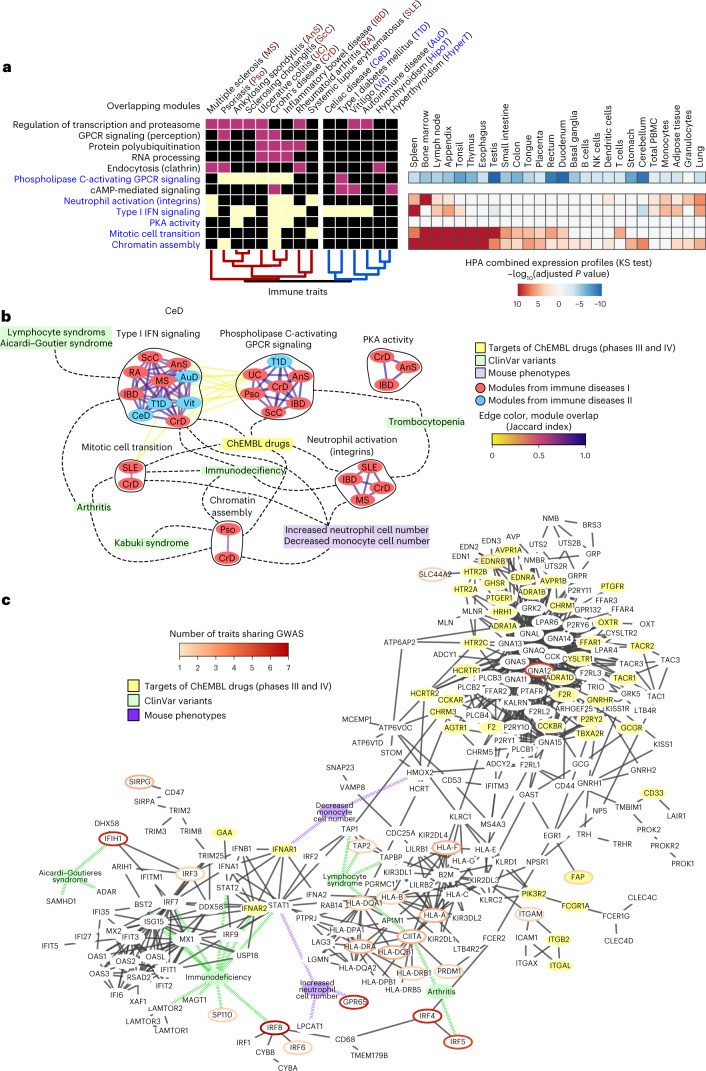
Gene module analysis of autoimmune diseases. **a**, Heatmap showing the overlap between gene modules across traits (color-coded as in Fig. 3a,c,d). The GOBP description is based on the results of a GOBP enrichment test (one-sided Fisher’s exact test, BH adjustment, Methods). The heatmap in the right-hand panel shows the gene set enrichment analysis carried out on the expression data from different tissues extracted from Human Protein Atlas (HPA) for the gene modules in blue (two-sided Kolmogorov–Smirnov test, Methods). After BH adjustment for multiple testing, the *P* value of the test was log transformed and given a positive value if the median distribution for the foreground was higher than the background and a negative value if it was lower. **b**, Shared modules as a network, nodes are gene modules associated with different immune-related traits colored blue or red for the two trait subgroups; edges represent a high degree of overlap at the gene level (Jaccard index > 0.7). Gene modules linked to different traits are given in black circles. Gene modules are linked with the yellow node ‘ChEMBL-drugs’ when they contain targets for drugs in clinical trials (phases III and IV, ChEMBL); linked with green nodes when they are enriched in genes with clinical variants for a given disease; and linked with purple nodes when they are enriched for the corresponding KO phenotypes (one-sided Fisher’s exact test, adjusted *P* < 0.05). **c**, Network corresponding to genes found in gene modules enriched for Type I interferon (INF) signaling, phospholipase C-activating GPCR signaling, neutrophil activation (integrins) and protein kinase A (PKA) activity. Edge filtering, node and edge colors are the same as in Fig. 3c,d.

Traits related to the immune system are well represented in our analysis, falling into three different groups: one cluster containing systemic and organ-specific diseases; one cluster of immune cell measurements; and a third, more heterogeneous, cluster (Fig. 3a and Supplementary Table 2). In Fig. 4a we represent the first of these clusters, which can be further subdivided into a subgroup linking IBD, multiple sclerosis and systemic lupus erythematosus, and one linking celiac disease, vitiligo and other diseases. We found six gene modules that are specifically enriched with at least one of these two groups of traits, including gene modules related to GPCR signaling, neutrophil activation and interferon signaling. Genes present in these modules show higher relative expression (Fig. 4a, right) in key immune tissues.

The six gene modules are shown in Fig. 4b with a connection between them when there is a significant gene-level overlap (Fig. 4b; Methods). For representation (Fig. 4c), we selected genes from modules linked with at least three immune-mediated diseases and kept a subset of interactions of high confidence (Methods). We found multiple genes with ClinVar variants from patients with primary immune deficiencies (for example, *IRF9*, *IRF7*, *STAT1*, *STAT2*) that are not GWAS-linked genes but are in their network vicinity, providing evidence of the importance of this gene module for these diseases.

To pinpoint drugs with repurposing potential, we excluded those targeting diseases in the same therapeutic areas as the immune-mediated group of diseases, identifying 49 drugs with 20 targets. These include ulimorelin, an agonist of the ghrelin hormone secretagogue receptor *GHSR* used to treat gastrointestinal obstruction. Ghrelin hormone signaling has been studied in the context of age-related chronic inflammation^33^, psoriasis^34^ and IBD (reviewed in ref. ^35^) indicating a potential repurposing opportunity. The 49 drugs with repurposing potential are listed in Supplementary Table 3 with information on target genes and clinical trials.

### Network-assisted candidate gene prioritization for IBD

Although the gene modules we have described can highlight biological pathways shared between genetically related traits, identifying causal genes at individual GWAS loci is important for prioritizing therapeutic targets. Existing methods such as GRAIL^36^, DEPICT^37^ and MAGMA^38^ prioritize genes based on biological pathways but do not fully use genome-wide protein interaction networks, which can provide finer-grained information over GO terms.

Here, we use network propagation to prioritize genes at IBD GWAS loci, similar to our previous work on Alzheimer’s disease^39^. We used two alternative methods of defining seed genes for the network. First, we manually curated 37 genes with high confidence of being causally related to either Crohn’s disease or ulcerative colitis (Supplementary Table 4) and second, we used the Open Targets L2G score to automatically select 110 genes with L2G > 0.5 at established IBD loci^40,41^ (Methods and Supplementary Table 4). To obtain network propagation scores, we compared each gene’s score with 1,000 runs using the same number of randomly selected input genes, to give the PPR percentile value (Methods). We obtained unbiased network propagation values for each seed gene by excluding them one at a time (Methods).

**Fig. 5:**
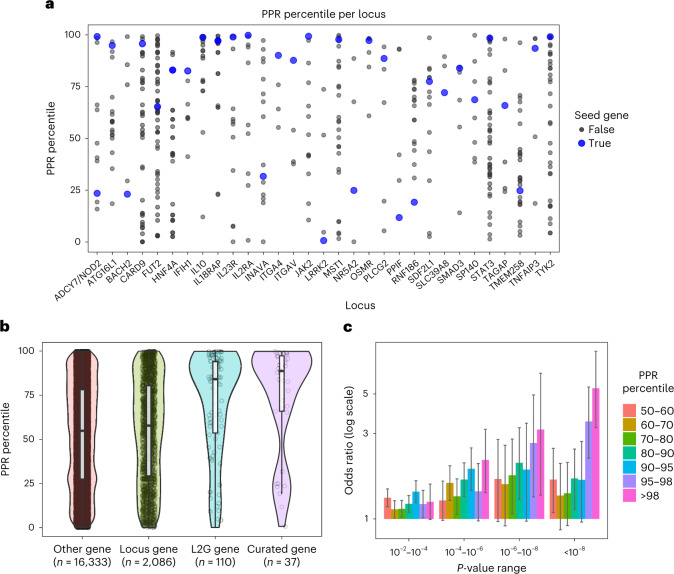
An IBD-specific network is enriched for likely causal genes. **a**, Curated IBD seed genes (*N* = 37) tend to have a higher network propagation score (PPR percentile) than other genes within 200 kb at the same loci. **b**, Genes selected by high Open Targets L2G score also tend to have high PRR percentile, highlighting network evidence as complementary to typical locus features. In the boxplots, the middle lines represents the median, the limits of the box are quartiles 1 and 3 and the whiskers represents 1.5× the interquartile range. **c**, Genome-wide, genes with low *P*-value SNPs within 10 kb are enriched for high PPR percentile (one-sided Fisher’s exact test). Data are presented as the mean ± s.d.

The curated seed genes had far higher network scores than other genes within 200 kb (*P* = 7.4 × 10^−6^, one-tailed Wilcoxon rank sum test), indicating that most seed genes have close interactions with other seed genes (Fig. 5a). The same was true when considering seed genes exclusively in the L2G gene set (Fig. 5b; *P* = 3 × 10^−10^, one-tailed Wilcoxon rank sum test), indicating that many of these are also strong IBD candidate genes. Finally, we examined the enrichment of low SNP *P* values within 10 kb of genes having high network scores. This revealed a progressive enrichment of low *P* values near genes with higher network scores (Fig. 5c), which held for the large number of genes linked to SNPs not reaching the typical genome-wide significance threshold of 5 × 10^−8^ for locus discovery.

Curated genes with strong network support include the drug targets *TYK2, ICAM1* and *ITGA4*, and *NOD2* and *IL23R*, which have missense variants implicating them as modulators of IBD^42–44^. A small number of curated genes had lower network support, which could be due to these genes affecting IBD via pathways distinct from the biological functions covered most well by the curated gene set. Across IBD loci without curated genes, our network scores rank 42 candidates as being more highly functionally connected than the remaining genes at the locus (Supplementary Table 4 and Methods). Although many of these were already strong IBD candidate genes, some have found strong support only recently. A clear example is the *RIPK2* locus. Although *OSGIN2* is nearest to IBD lead SNP rs7015630 (38 kb distal), it has no apparent functional links with IBD (network score 43%). By contrast, *RIPK2* (108 kb distal, network score 99%) encodes for a mediator of inflammatory signaling via interaction with the bacterial sensor *NOD2* (ref. ^45^). Network information can also provide a comparison point for other evidence sources. At the *DLD-SLC26A3* locus, there is moderate evidence of genetic colocalization between IBD and an expression quantitative trait loci (eQTL) for *DLD* in various tissues (Open Targets Genetics portal). However, DLD has no clear functional links with IBD and receives a low network score (14%). By contrast, *SLC26A3* is a chloride anion transporter highly expressed in the human colon, with a high network score (98.4% in the L2G seed gene network), and its expression has been recently associated with clinical outcomes in ulcerative colitis^46^. IBD candidate genes that have high network scores but have not been well characterized in the context of IBD include *PTPRC* (a phosphatase required for T cell activation) and *BTBD8*, which is functionally connected to autophagy by the network analysis (via *WIPI2* and *ATG16L1*).

To study the pleiotropy of the curated and candidate genes we looked at the eight gene modules linked by our analysis to IBD (Supplementary Fig. 8). Of the 37 curated and 42 candidate genes, 35 (14 curated and 21 candidate) are found within these modules. Interestingly, we found that most of these genes are in modules that are only linked to IBD; in particular, a module that is enriched for genes related to receptor signaling via the JAK–STAT pathway (Supplementary Fig. 9). Conversely, the most pleiotropic modules linked to IBD have very few IBD candidate genes within them. As expected, these pleiotropic modules tend to be associated with traits that are related to the immune system, with the exception of the most pleiotropic module, which is enriched for genes related to protein ubiquitination (Supplementary Fig. 8). This analysis suggests that the JAK–STAT-related module is likely to be the best source of novel candidate disease genes and drug targets that are more inclined to be specific to IBD.

## Discussion

We identified gene modules associated with 906 human traits, taking advantage of the increased coverage of human interactome mapping and novel tools for SNP-to-gene mapping^11^. As seen in other studies^9^, network expansion can retrieve previously known disease genes not identified by GWAS, including those not in GWAS loci but that may modulate the same biological processes. Even when excluding genes with direct genetic support, such interacting genes are enriched for successful drug targets^12^. Genes identified by network expansion will not have information on the direction of effect and additional work and interpretation are needed to gain insights into the direction of impact of modulating such genes. Although there are several algorithms to perform network propagation, recent studies have shown that they tend to perform similarly^47^ and the network used has a stronger impact on performance^9^. For this reason, improvements in mapping coverage and computational or experimental approaches to deriving tissue- or cell-type-specific networks^8^ could have a large impact on the future effectiveness of network expansion.

We showed examples of disease-linked gene modules that were also enriched in genes carrying clinical variants for the same or related diseases. In many cases, genes with clinical variants did not overlap with the GWAS-linked genes, which is likely due to a lower frequency of clinical variants. Testing for burden of loss-of-function variants within selected gene sets is an approach used to study the impact of low-frequency variants^48,49^ and we suggest that the gene modules identified here could be ideally suited for this purpose. The gene modules identified here relate to specific aspects of cell biology with different human traits. Analysis of mouse phenotypes and ClinVar variants provided additional evidence for some of the identified relationships. Additional experimental work, in particular with appropriate models (for example, organoids, mouse models), is needed to follow up on some of the derived associations. Beyond identifying gene modules, our GWAS-based network approach can also be used to prioritize disease genes at individual loci by their role within specific biological processes, as we showed for IBD.

The most pleiotropic gene modules share some aspects of cell biology that have been defined as highly pleiotropic in gene deletion studies of yeast^15^. Gene modules linked with different traits could provide opportunities for drug repurposing or cross-disease drug development. However, targeting pleiotropic processes could raise safety concerns. We find that these modules are enriched for genes that are ubiquitously expressed, and have many gene deletion phenotypes and a higher number of genetic interactions. However, we do not find a simple correlation between the number of traits associated with a gene module and these metrics. This may suggest that some highly pleiotropic processes may be safe to target or that metrics such as CRISPR deletion phenotypes and ubiquitous expression may be insufficient to judge drug target safety.

Comparing the pleiotropy of cellular processes as defined by module–trait associations with that defined by gene deletion studies suggests that, although there are some similarities, gene deletion studies tend to miss pleiotropy that relates to cell-to-cell communication. This is not surprising given that CRISPR screens in cell lines typically assay for phenotypes measured in single cells. Conversely, our trait-to-module analysis tends to miss pleiotropy that is highly essential to cells. We suggest that (some of) these essential cellular processes may be lethal if genetically perturbed, and therefore associated variants are not observed in human populations and not seen in genetic association studies.

Interestingly, traits that are linked with highly pleiotropic gene modules tend to have a larger number of starting GWAS seed genes, which usually have larger sample sizes. This suggests that the larger the number of loci linked to a trait, and likely greater sample sizes, the higher the chances that this trait will be genetically linked to highly pleiotropic biological processes. Although it has been suggested that the heritability of complex traits is broadly spread along the genome^16^, our analysis indicates that, across a large number of traits, this heritability overlaps in a nonrandom fashion.

In summary, network expansion of GWAS is a powerful tool for the identification of genes and cellular processes linked to human traits, and application in multitrait analysis can reveal pleiotropy of human biological pathways at the level of the organism, as well as highlight new opportunities for drug development and repurposing.

## Methods

### Human interactome, GWAS traits and linked genes analyzed

We created a comprehensive human interactome, merging an interactome developed for the Open Targets (www.opentargets.org) project (version from November 2019), with STRING v.11.0. The Open Targets Interactome network was constructed during this project and contains human data only, including physical interaction data from IntAct, causality associations from SIGNOR and binarized pathway reaction relationships from Reactome. More details about the network construction can be found in the Supplementary Information and at https://platform-docs.opentargets.org/target/molecular-interactions. STRING functional interactions were human only and selected to have a STRING edge score ≥0.75. All identifiers were mapped to Ensembl gene identifiers and, after removing duplicated edges and self-loops, the final network contained 18,410 nodes and 571,917 edges.

### Network propagation of GWAS-linked genes

From a total of 1,221 traits, we selected 1,002 mapped to EFO terms (www.ebi.ac.uk/efo/) included in the Open Targets genetic portal, with at least two genes mapped to our interactome with a L2G score of 0.5 or above (defined as seed nodes). The network-based approach was run individually for each trait, with each protein having a weight corresponding to the L2G score (between 0.5 and 1.0). The input was diffused through the interactome using the PPR algorithm included in the R package igraph (v.1.2.4.2). To generate the modules, we selected nodes with a PPR ranking score greater than the third quartile (Q3, 75%) and performed walktrap clustering (igraph v.1.2.4.2). When the number of nodes in one module was >300, we repeated the clustering inside this community until all resulting clusters were <300 genes. To define gene modules as significantly associated with a trait, we used a Kolmogorov–Smirnov test to determine whether ranks (based on PPR) of genes in a module were greater than the background ranks of all the nodes considered for the walktrap clustering. We tested only modules with at least ten genes and where two or more of them were seed genes (L2G > 0.5), and we corrected the resulting *P* values for multiple testing using BH adjustment. On the basis of this, we identified a total of 2,021 associations between a gene module and a trait.

### Benchmarking the capacity to predict disease-associated genes from the network expansion

To benchmark both the predictive power of the ranking score resulting from the PPR and the genetic portal data when compared with a GWAS catalog (https://www.ebi.ac.uk/gwas/; based on gene proximity), we computed ROC curves using as true positives the genes linked to diseases from the Jensen lab DISEASE database (diseases.jensenlab.org). This database provides a score measuring this association; benchmarking was done using five different score thresholds (DIS0, all genes; DIS1, score >25%; DIS2, score >50%; DIS3, score >75%; and DIS4, maximum value for the score). We calculated the ROC curves and the area under the ROC curve (AUC) for traits with at least ten true positives. Also, we randomized both nodes in the network (keeping the degree distribution) as well as the true positives 1,000 times each. We then calculated the AUC values and the subsequent *Z-*scores. As an extra benchmark, we used the clinical trial data contained in ChEMBL (https://www.ebi.ac.uk/chembl/), considering as true positives drug targets tested for a certain disease at clinical phase II or higher.

### Trait–trait relationships defined by the similarity of the network propagation

We calculated the Manhattan distance between the 1,002 traits using the full PPR ranking score, followed by hierarchical clustering, resulting in 54 clusters (height distance = 1). To further characterize the trait clusters, we selected those having at least five traits, obtained their EFO ancestry and calculated their frequency per cluster. The highest frequency per cluster is used to define nine groups color-coded in Fig. 2a. To complement the description of clusters belonging to the most general group ‘measurement’ and ‘material property’, we extracted EFO ancestry terms using manually assigned terms from the EFO ancestry with a lower frequency (Fig. 2a). The ChEMBL database (https://www.ebi.ac.uk/chembl/) was used to calculate the counts of both drugs and drug targets for each of the trait clusters, using information for drugs in clinical trials phases III and IV. To further illustrate the validity of this approach, we selected three trait clusters (Fig. 2b) as examples of valid trait-to-trait relations.

### Multitrait gene module analysis

Significant modules identified for each trait (described above) were compared across traits by measuring the overlap in genes using the Jaccard index. Gene modules with a Jaccard index ≥0.70 were considered common across two traits. From the 2,021 pairs of gene module–trait associations, 886 are unique to a single trait and the remainder can be collapsed (that is, considered highly overlapping or the same gene module). This results in 73 gene modules that are enriched in network propagation signals for two or more traits. To identify subgroups of related traits, we clustered those linked to the 73 multitrait modules on the basis of the Manhattan distance of their full PPR ranking score (as above) using hierarchical clustering. Subgroups were defined with a height cutoff of 0.7 and we identified gene modules that were more specific to each subgroup of traits using a one-sided Fisher’s exact test and BH multiple testing correction. We retained trait subgroups with at least three traits and a significant presence of at least one group of overlapping modules.

### Relating pleiotropy from GWAS module with gene expression and deletion phenotypes

We used the BioGRID Open Repository of CRISPR Screens (ORCS, v.1.1.11, https://orcs.thebiogrid.org/), which contains 1,342 studies measuring the impact of gene deletions on viability and other cellular measurements, including cell-cycle progression, response to different stresses, transport and others. On the basis of these CRISPR screens, we defined as pleiotropic those genes that had a cell-based phenotype in more than half of the screens. We defined genes likely to be expressed in many tissues as those having an expression level above the median for a given tissue in more than half of the tissues in the Human Protein Atlas (https://www.proteinatlas.org/). To compare the enrichment of genes defined as highly pleiotropic in our analysis with those defined by CRISP studies, we performed an enrichment analysis for each GOBP term using a Gene Set Enrichment Analysis test (cluster profiler package, v.4.2.2).

### Gene module annotations and enrichment analysis

The gene KD mouse phenotypes were extracted from the International Mouse Phenotyping Consortium (https://www.mousephenotype.org/) and the clinical variants were extracted from the ClinVar database (National Center for Biotechnology Information (NCBI), https://www.ncbi.nlm.nih.gov/clinvar/). For the enrichment of genes from clinical variants, diseases were grouped into larger categories. For the enrichment of genes from clinical variants referred to in Figs. 3c,d and 4b,c, we downloaded data from ClinVar (NCBI), filtered out all benign associations and grouped the phenotypes into higher categories as follows: tooth agenesis (tooth agenesis, selective tooth agenesis 4, 7 and 8); bone-related diseases (sclerosteosis 1, osteoarthritis, osteopetrosis, osteoporosis, osteogenesis imperfecta and osteopenia); asthma (asthma and nasal polyps, susceptibility to asthma and asthma-related traits, diminished response to leukotriene treatment in asthma, asthma and aspirine intolerance); autoimmune condition (familial cold autoinflammatory syndromes); immunodeficiency (immunodeficiency due to a defect in MAPBP-interacting protein, hepatic veno-occlusive disease with immunodeficiency, immunodeficiency-centromeric instability-facial anomalies syndrome 1, immunodeficiency 31a, 31C, 32a, 32b, 38, 39, 44 and 45, immunodeficiency X-linked, with magnesium defect, Epstein–Barr virus infection, and neoplasia, combined immunodeficiency, severe T cell immunodeficiency and immunodeficiency 65 with susceptibility to viral infections); lymphocyte syndrome (bare lymphocyte syndrome types 1 and 2); arthritis (rheumatoid arthritis and juvenile arthritis); Kabuki syndrome (Kabuki syndrome 1 and 2); thrombocytopenia (thrombocytopenia, dyserythropoietic anemia with thrombocytopenia, GATA-1-related thrombocytopenia with dyserythropoiesis, X-linked thrombocytopenia without dyserythropoietic anemia, thrombocytopenia with platelet dysfunction, hemolysis, imbalanced globin synthesis, radioulnar synostosis with amegakaryocytic thrombocytopenia 2 and macrothrombocytopenia); anemia (anemia, dyserythropoietic anemia with thrombocytopenia, aplastic anemia, CD59-mediated hemolytic anemia with or without immune-mediated polyneuropathy and Diamond–Blackfan anemia); and Aicardi–Goutieres syndrome (Aicardi–Goutieres syndrome 4, 6 and 7).

### IBD network analyses for fine-mapping

To identify robust IBD-associated loci, we extracted loci defined in the Open Targets Genetics portal (genetics.opentargets.org) for two IBD GWAS^40,41^. Because each GWAS may identify different lead variants, we merged loci defined by lead variants within 200 kb of each other. We extracted the L2G score reported for all genes at each locus, and for merged loci we took the average L2G score for each gene across the loci. We curated 37 high-confidence IBD genes on the basis of the presence of fine-mapped deleterious coding variants, genes whose protein products are the targets of approved IBD drugs and the literature. We defined additional seed gene sets by selecting the top gene at each locus that had an L2G score >0.5. We ran network propagation as described in the Results section of the main text. However, to obtain unbiased scores for seed genes themselves, we left each seed gene out of the input in turn, and ran network propagation to obtain a score based on the remaining *N* *−* 1 seed genes. To compute the PPR percentile for seed genes, we used the PPR percentile from the single network propagation run in which that seed gene was excluded from the input. For all other genes, we used the median PPR percentile across *N* seed gene runs. The plots in Fig. 5 are based on PPR percentiles from the curated seed gene network. To assess the enrichment of low *P* value SNPs near high network genes (Fig. 5c), we first determined for each gene the minimum *P* value among SNPs within 10 kb of the gene’s footprint based on IBD GWAS summary statistics from de Lange et al.^41^. We used Fisher’s exact test to determine the odds ratio for genes with a high network score (in each defined bin) having a low minimum SNP *P* value, relative to genes with low network scores (PPR percentile <50).

PPR percentiles discussed in the text are the average for each gene across the curated and L2G > 0.5 networks. We identified IBD candidate genes that stand out on the basis of their network score (Supplementary Table 4) by selecting all locus genes that had an average PPR percentile >90 and L2G > 0.1, and where no other gene at the same locus had PPR percentile >80 and L2G > 0.1.

### Statistics and reproducibility

Data collection and analysis were not blind to the conditions of the experiments. Sample sizes (*n*) are indicated in the figure or figure caption when appropriate. No statistical method was used to predetermine sample size, but where appropriate sample size was considered in statistical tests. No data were excluded from the analyses and the experiments were not randomized.

### Ethics statement

No ethical approval was required for this work.

### Reporting summary

Further information on research design is available in the Nature Portfolio Reporting Summary linked to this article.

## Online content

Any methods, additional references, Nature Portfolio reporting summaries, source data, extended data, supplementary information, acknowledgements, peer review information; details of author contributions and competing interests; and statements of data and code availability are available at 10.1038/s41588-023-01327-9.

## Supplementary information

Supplementary Information Supplementary Figs. 1–9

Reporting Summary

Supplementary Data 1 List and annotations of the 1,002 traits studied and their clustering by network propagation scores.

Supplementary Data 2 Gene modules linked to each trait and their annotations.

Supplementary Data 3 Detailed gene and gene module information for examples in Figs. 3c,d and 4c.

Supplementary Data 4 IBD candidate gene information.

## Data Availability

All data generated or analyzed during this study are included in this published article (and its Supplementary Information files). Publicly available repositories can be accessed as follows: OTAR interactome (ftp://ftp.ebi.ac.uk/pub/databases/intact/various/ot_graphdb/current), STRING v.11.0 (https://string-db.org/), Open Targets Genetics portal (g), Mouse KO phenotypes (IMPC, https://www.mousephenotype.org/), ClinVar (NCBI, https://www.ncbi.nlm.nih.gov/clinvar/), BioGRID Open Repository of CRISPR Screens (ORCS, v.1.1.11, https://orcs.thebiogrid.org/), BiGRID v.4.4.202 for protein and genetic interactions (https://thebiogrid.org/), Human Protein Atlas (https://www.proteinatlas.org/), DISEASE database (https://diseases.jensenlab.org) and ChEMBL (https://www.ebi.ac.uk/chembl/).

## Code availability

The network-based method and all subsequent analysis was performed using R software (v.4.0.2) as described in the Methods, combined with the following packages: igraph (v.1.2.4.2, for Personalized PageRank and walktrap clustering), pROC (v.1.16.2, for ROC curves and AUCs calculations when applicable), clusterprofiler (v.4.2.2, for GOBP enrichment analysis in the description of the modules as well as GSEA test), pheatmap (v.1.0.12, for heatmap calculations when applicable), ggplot2 (v.3.3.2 for Fig. 5), vioplot (v.0.3.5 for violin plots) viridis (v.0.3.0) and RColorBrewer (v.1.1.2) both for color palette generation. The R functions used to perform the network expansion (Propagation using PPR and community detection to define gene modules) are publicly available in Zenodo (10.5281/zenodo.7575743).
