# Supplementary material for: Network expansion of genetic associations defines a pleiotropy map of human cell biology
Source: Nat Genet. Author manuscript; Available in PMC 2023 Mar 15. (PMC10011132; doi:10.1038/s41588-023-01327-9)
Supplement: Additional Supplementary Files [file EMS163741-supplement-Additional_Supplementary_Files.pdf]

1. Supplementary Information:

A. Flat Files

Item	Present?	Filename<br>This should be the name the file is saved as when it is uploaded to our system, and should include the file extension. The extension must be .pdf	A brief, numerical description of file contents.<br>i.e.: *Supplementary Figures 1-4, Supplementary Discussion, and Supplementary Tables 1-4.*
Supplementary Information	Yes	Supplementary_information.pdf	Supplementary figures 1-9
Reporting Summary	Yes	NG-A58046R2_Beltrao_RSf.pdf	

B. Additional Supplementary Files

Type	Number<br>If there are multiple files of the same type this should be the numerical indicator. i.e. "1" for Video 1, "2" for Video 2, etc.	Filename<br>This should be the name the file is saved as when it is uploaded to our system, and should include the file extension. i.e.: *Smith_ Supplementary_Video_1.mov*	Legend or Descriptive Caption<br>Describe the contents of the file
Supplementary Data	1	SupplementaryData1.xlsx	List and annotations of the 1002 traits studied and their clustering by network propagation scores
Supplementary Data	2	SupplementaryData2.xlsx	Gene modules linked to each trait and their annotations
Supplementary Data	3	SupplementaryData3.xlsx	Detailed gene and gene module information for examples in 3C, 3D and 4C
Supplementary Data	4	SupplementaryData4.xlsx	IBD candidate gene information
