# Supplementary material for: Network expansion of genetic associations defines a pleiotropy map of human cell biology
Source: Nat Genet. 2023 Feb 23;55(3):389–98. doi: 10.1038/s41588-023-01327-9 (PMC10011132; doi:10.1038/s41588-023-01327-9)
Supplement: Supplementary file 1 [file 41588_2023_1327_MOESM1_ESM.pdf]

nature genetics

Article

https://doi.org/10.1038/s41588-023-01327-9

Network expansion of genetic associations defines a pleiotropy map of human cell biology

In the format provided by the authors and unedited

Supplementary Information

Network expansion of genetic associations defines a pleiotropy map of human cell biology

Inigo Barrio-Hernandez[1,2], Jeremy Schwartzentruber[1,2,3], Anjali Shrivastava[1,2], Noemi del-Toro[1,2], Asier Gonzalez[1,2], Qian Zhang[3], Edward Mountjoy[1,2], Daniel Suveges[1,2], David Ochoa[1,2], Maya Ghoussaini[1,2], Glyn Bradley[4], Henning Hermjakob[1,2], Sandra Orchard[1,2], Ian Dunham[1,2,3], Carl A. Anderson[2,,3], Pablo Porras[1,2], Pedro Beltrao[1,2,5#]

1 – European Molecular Biology Laboratory, European Bioinformatics Institute (EMBL-EBI), Wellcome Genome Campus, Cambridge CB10 1SD, UK

2 – Open Targets, Wellcome Genome Campus, Cambridge, CB10 1SA, UK

3 – Wellcome Sanger Institute, Wellcome Genome Campus, Cambridge,CB10 1SA, UK

4 – Computational Biology, Genomic Sciences, GSK, Stevenage, UK

5 – Institute of Molecular Systems Biology, ETH Zürich, 8093 Zürich, Switzerland

# Correspondence to pbeltrao@ethz.ch

Supplementary figures

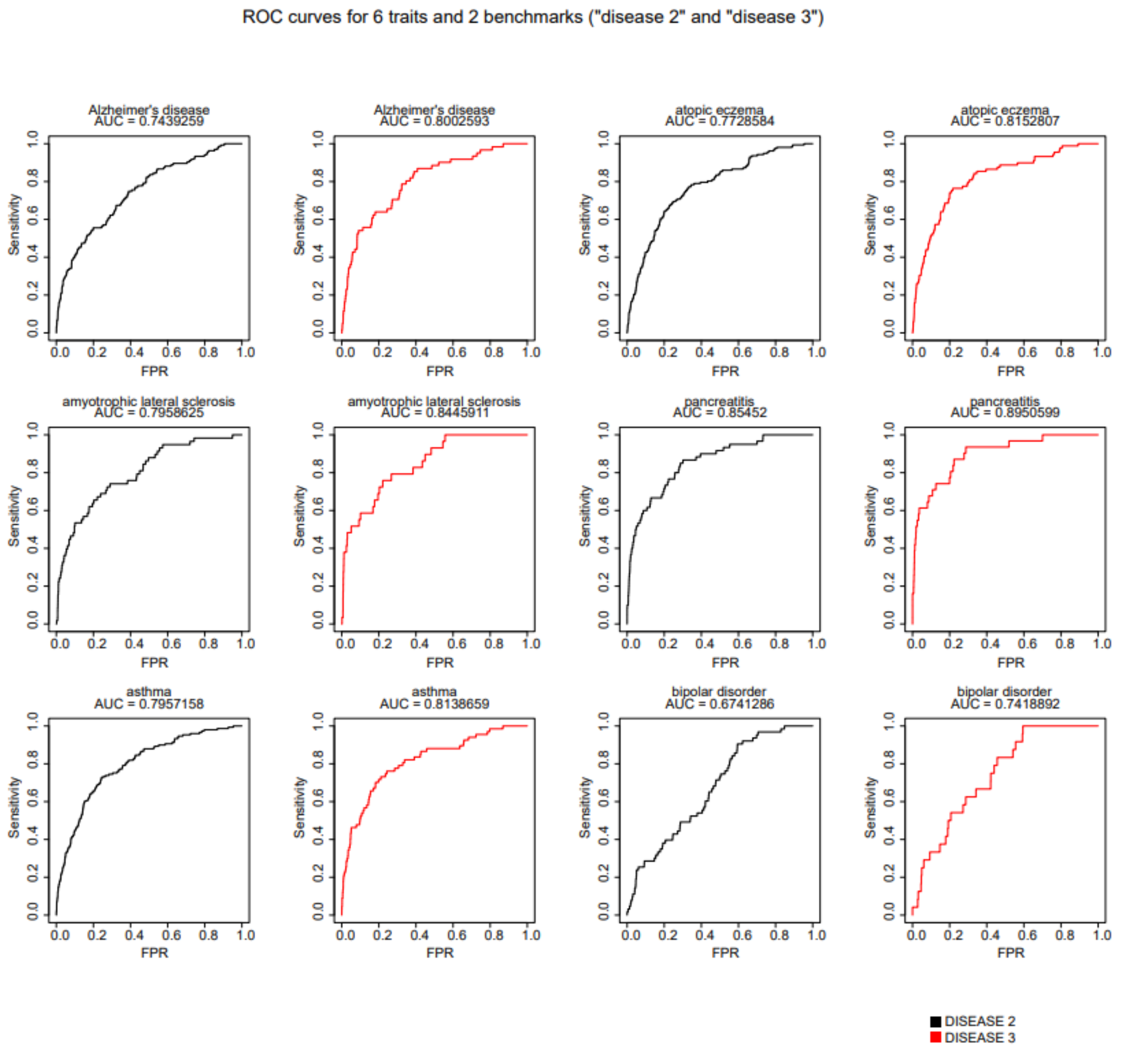

Supplementary Figure 1 - Example ROC curve analysis for prediction of known disease genes. As examples, we selected 6 traits with known disease genes listed in the DISEASE database with two confidence levels (level 2 and 3). For each trait and benchmark we calculated capacity of the network propagation scores to predict these disease genes. This is summarised here in the form of ROC curves.

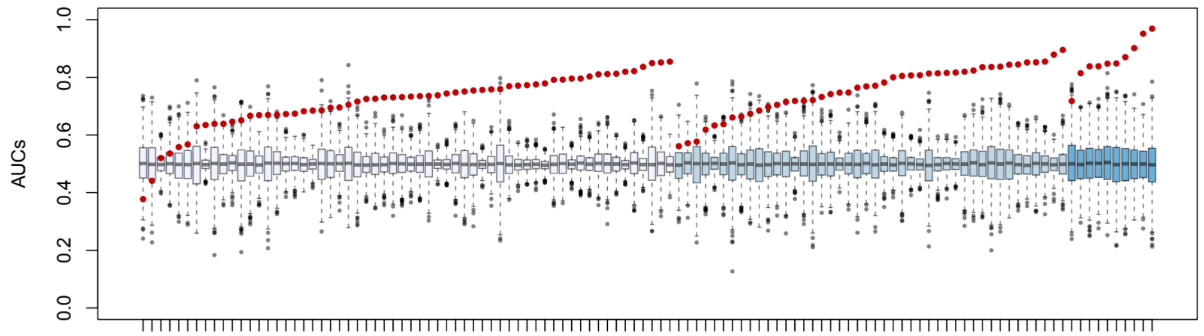

Supplementary Figure 2 - True positive randomization for prediction of known disease genes from network propagation of GWAS linked genes. Results of the True positive randomizations for disease associated genes from DISEASE subdivisions 2, 3 and 4 (increasingly dark colour of blue for each). The red dots correspond to the observed AUC and the boxplot corresponds to the distribution AUCs values obtained from performing network propagations after 1000 randomizations of the true positive set (n=1000).In the boxplots the middle lines represents the median, the limits of the box are the quartiles 1 and 3 and the whiskers represents 1.5 times the interquartile range. The black dots are values outside the whiskers (outliers)

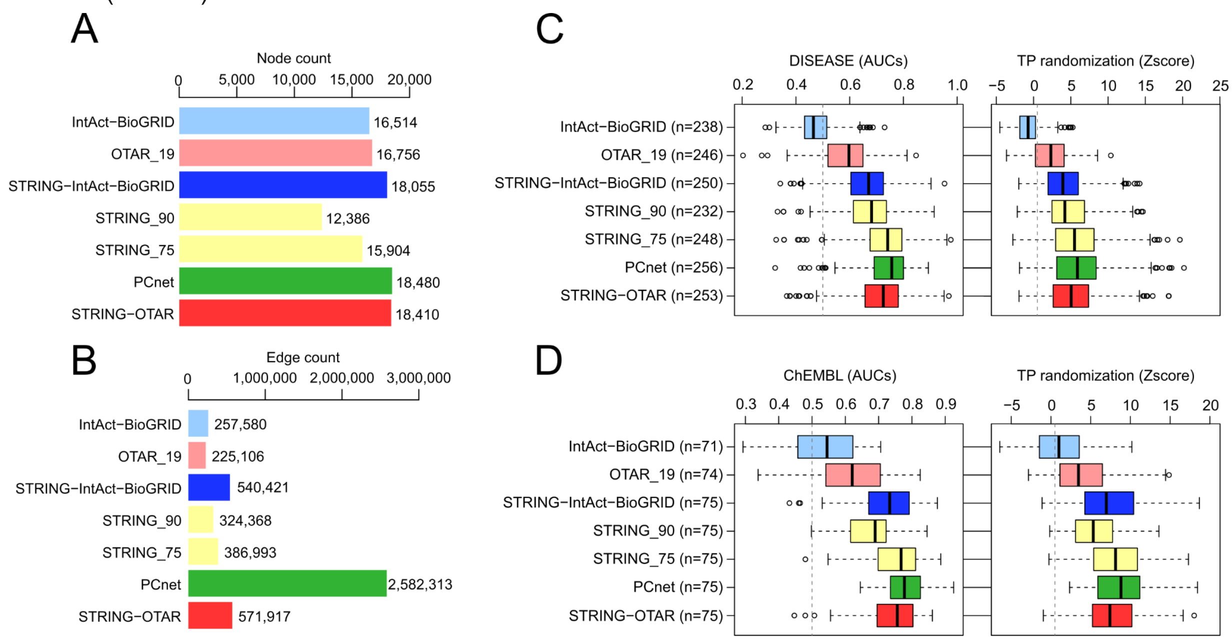

Supplementary Figure 3 - Comparison of different protein interaction networks. We tested the prediction of known disease genes or drug targets using the network expansion method based on different networks composed of subsets of the final composite network used in this study. These include the STRING interactions with a cutoff>0.75 and >0.9 (labelled STRING_75 and STRING_90); interactions compiled from high throughput studies (more than 1,000 interactions) deposited in either IntAct or BioGRID (labelled IntAct-BioGRID); the combined OpenTargets interactome (labelled OTAR_19, described in the manuscript); and combinations of the STRING interactions with the other two sets (labelled as STRING-IntAct-BioGRID and STRING-OTAR). Finally, we compared these also with the PCNet, a previously defined composite network. The size of each network is shown as the number of nodes (A) and edges (B). C) The performance of the network expansion in recovering the known disease associated genes that are not directly found by GWAS with the evaluation as the Area Under the ROC Curve (AUC) or Z-scores of this value relative to the permutation of the true positive sets.In the boxplots the middle

lines represents the median, the limits of the box are the quartiles 1 and 3 and the whiskers represents 1.5 times the interquartile range. The black dots are values outside the whiskers (outliers) D) The same as in C) but predicting known drug-targets.

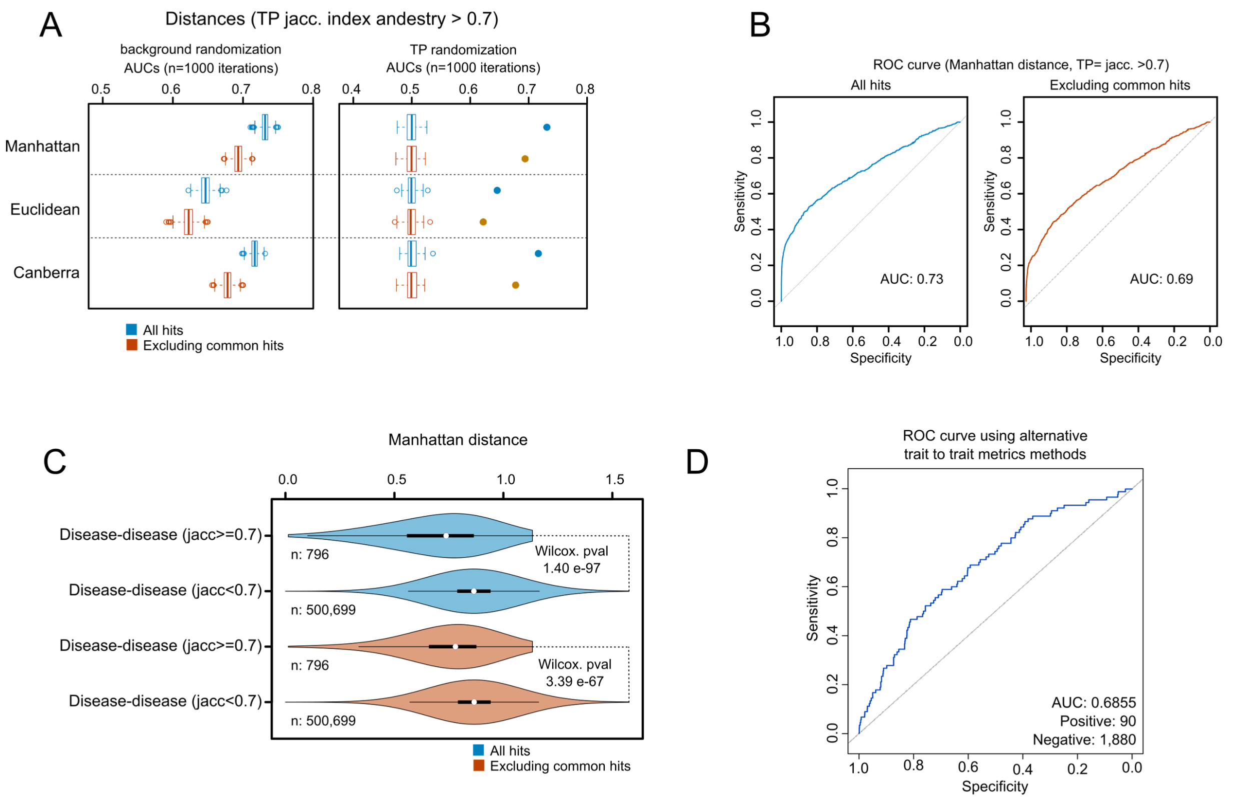

Supplementary figure 4 - Benchmark for predicting trait-trait similarity based on the network propagation scores. A) Areas under the ROC curve (AUCs) for three different disease-disease distance metrics: Manhattan, Euclidean and Canberra distances. They were calculated using the full PPR ranking scores after the network expansion for all disease-disease pairs. We considered as true positives the 796 trait-trait pairs with common ancestry (Jaccard score of ancestry terms from EFO annotation bigger or equal to 0.7). Using these annotations we defined 796 pairs of traits that are functionally related and therefore likely to have a common genetic basis (see Methods). To calculate the ROC curves, we sampled 1000 pairs from the negative space for 1000 iterations, the resulting AUCs were plotted in the boxplots. Similar distances and AUCs were calculated by running network propagation after excluding shared starting seed genes for a given pair of traits (Red, excluding common hits). The boxplots under TP randomization show the observed distribution of AUCs after randomization of the true-positive pairs of traits. In the boxplots the middle lines represent the median, the limits of the box are the quartiles 1 and 3 and the whiskers represent 1.5 times the interquartile range. The black dots are values outside the whiskers (outliers) B) Example of one of the ROC curves for Manhattan distance for the network propagation of all seed genes (All hits, AUC= 0.73), or after excluding common starting seed genes for a pair of traits (Excluding common hits, AUC=0.69) C) Violin plot showing the Manhattan distance distribution for all disease-disease pairs with shared ancestry (jaccard index >=0.70) considered as true positive and for all pairs considered as negative space. The Wilcoxon rank sum test (one-sided) was

calculated to measure the difference between both distributions. The propagations were run with all seed genes (blue) or excluding common seed genes for pairs of traits (red) In the violin plot, the white dots represent the median, the limits of the thick line correspond to quartiles 1 and 3 (25% and 75% of the distribution) and the limits of the thin line are 1.5 times the interquartile range. D) A benchmark dataset of related (90) and unrelated (1880) pairs of traits were collected from SNP-based approaches (see Methods). For each pair the similarity of their network propagation scores (Manhattan distance) was calculated and used to discriminate the positive from the negative set. AUC is the area under the ROC curve.

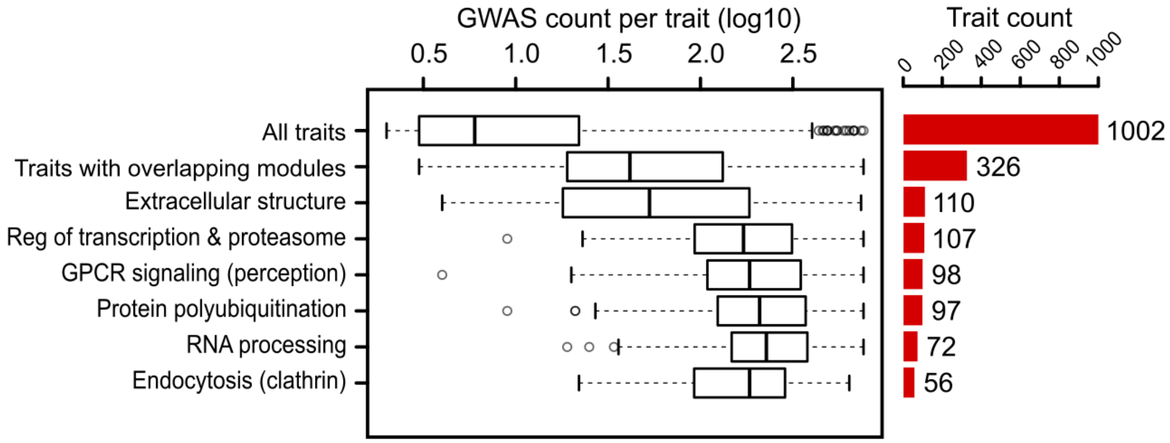

Supplementary figure 5 - . Boxplot showing the number of starting GWAS hits per trait (GWAS count) for all traits, for traits with shared modules and for traits that have highly pleiotropic modules (top 6, description based on GOBP annotation). In the left panel, barplot showing the total number of traits for each selection. In the boxplots the middle lines represent the median, the limits of the box are the quartiles 1 and 3 and the whiskers represent 1.5 times the interquartile range. The black dots are values outside the whiskers (outliers)

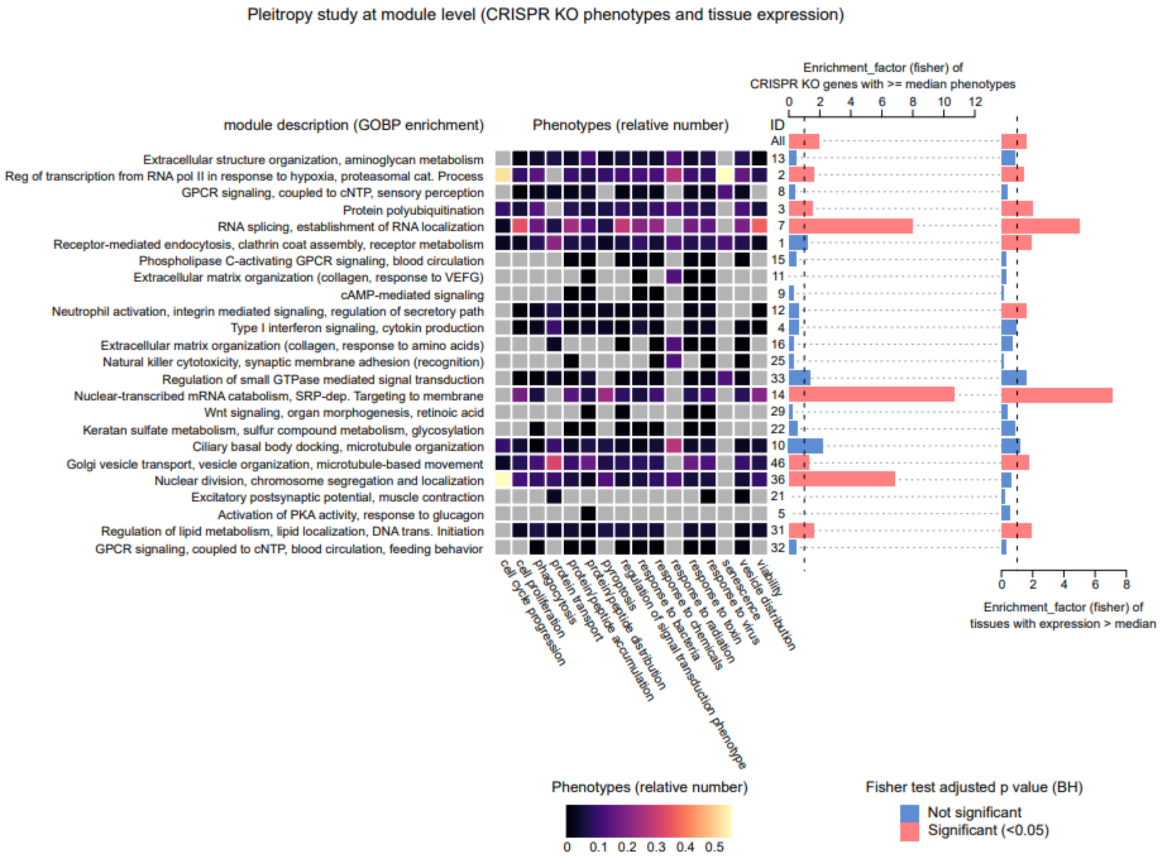

Supplementary Figure 6 - Relation between module-trait pleiotropy, ubiquity of gene expression and genes with large number of deletion phenotypes. Each row of the heatmap corresponds to one of several highly pleiotropic gene-modules that are ordered based on the number of linked traits (greater to lower) with a functional label based on a GOBP enrichment description (left side of the heatmap) and their ID (right side of the heatmap). The first row named “all” corresponds to the analysis of all genes contained in modules shared among at least 10 traits. The heatmap represents the number of CRISPR gene deletion phenotypes, for a given phenotype (shown in the x-axis labels) relative to all genes in the module. In the right panel the first barplot shows the enrichment factor (One- sided Fisher exact test, if the BH adjusted p-value is smaller than 0.05, the bar is colored in red) of genes with a number of CRISPR KO phenotypes bigger than the median, for each group of shared modules. The second barplot shows a similar metric for enrichment of genes highly expressed across tissues.

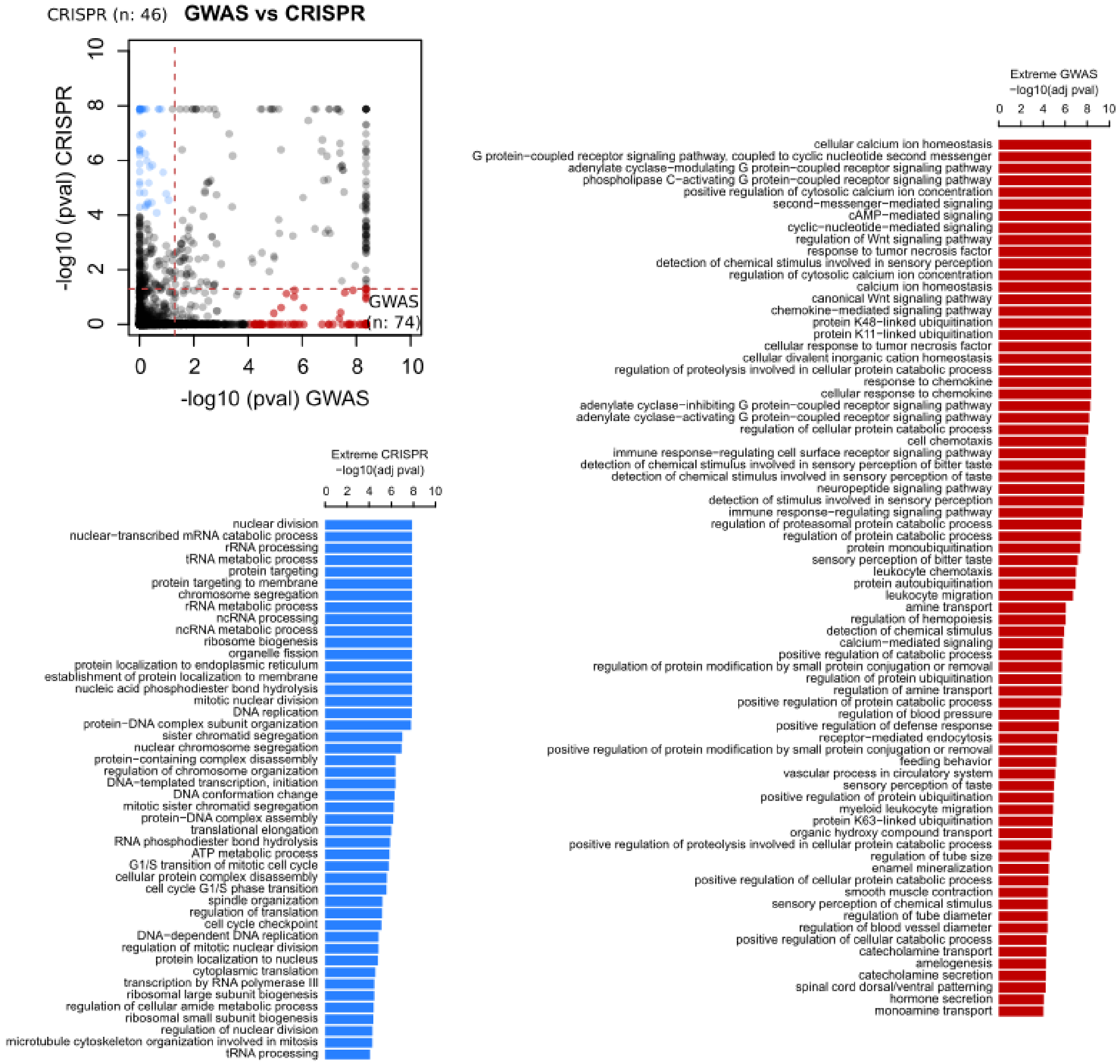

Supplementary Figure 7 - Comparing pleiotropy based on module-trait associations with that defined by CRISPR gene deletion studies. A) Scatter plot correlating the enrichment of each Gene Ontology (GO) Biological Process (BP) term in genes genes defined as pleiotropic using the number of CRISPR phenotypes (CRISPR) or the number of traits linked to a module carrying the genes (GWAS). The enrichment was performed using a Gene Set Enrichment Analysis (GSEA, one-sided) test and the plotted values are the -log(pvalue). Red dots represent GOBP terms that are not significant in the CRISPR group but that have an adjusted pvalue smaller than 0.0001 in the GWAS group. Blue dots represent the opposite (highly significant in CRISPR but not in GWAS). B and C) Significant and specific GOBP terms are shown the barplots matching their colors (-log10 BH adjusted pvalue, GSEA one-sided).

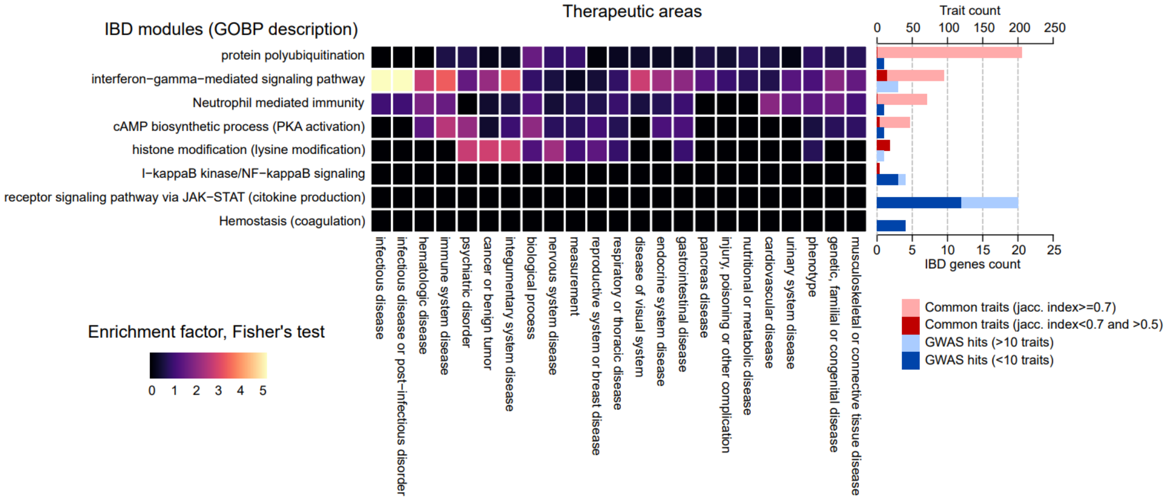

Supplementary Figure 8 - IBD linked modules and mapped curated/candidate IBD genes. Each row of the heatmap is one module linked to IBD. The modules are ranked by the total number of traits linked to the module (larger to smaller). The modules towards the bottom are only linked to IBD. The module's names on the left side of the heatmap are based on GOBP enrichment terms. The barplot in the right side of the heatmap has counts for the number of traits linked to these modules (top legend, red gradient bars) and the number of IBD candidate/curated genes within these modules (blue gradient bars). The values represented in the heatmap are the enrichment factor of therapeutic areas for the traits linked to these modules.

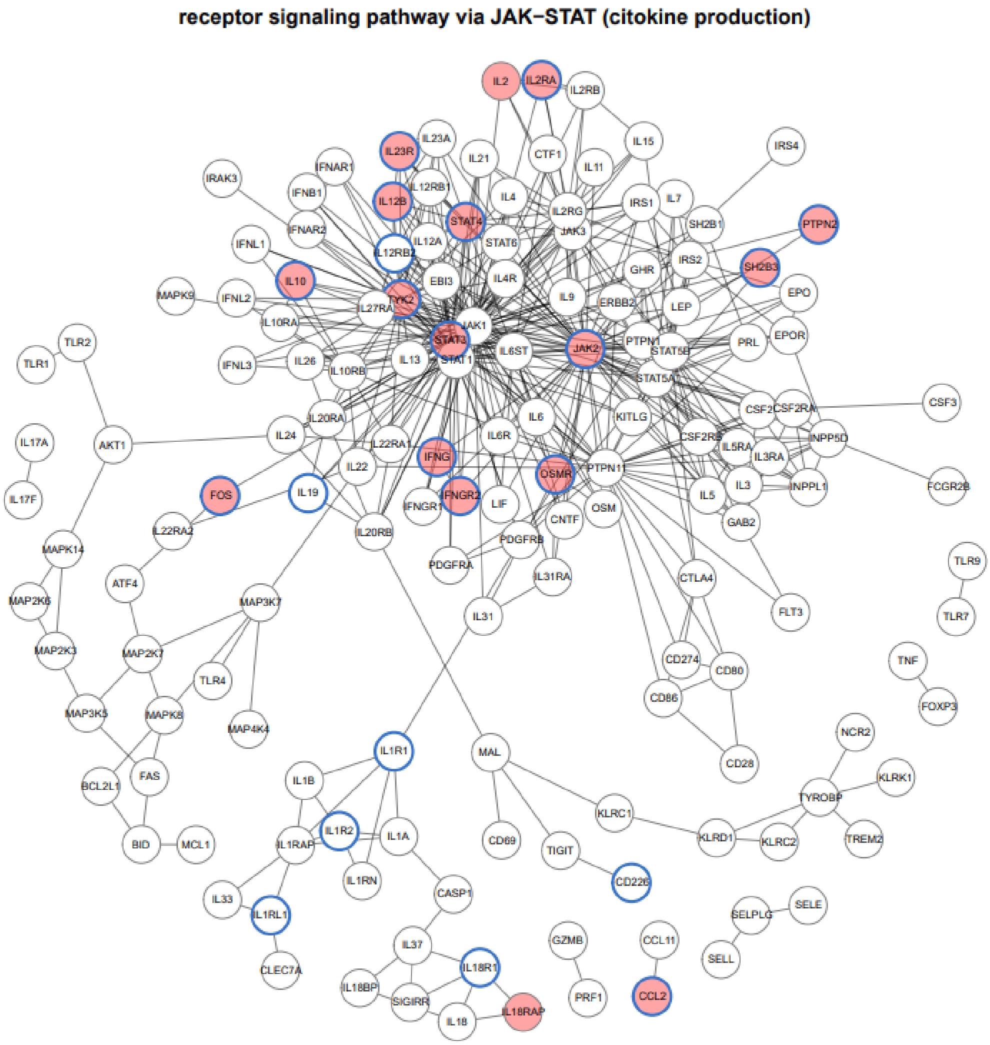

Supplementary Figure 9 - IBD module linked to JAK-STAT. Network showing the shared modules described as “receptor signalling pathway via JAK-STAT (cytokine production)”, edges filtered based on IntAct protein protein interactions. The nodes that are either candidate or curated IBD genes are colored in red , the blue border corresponds to genes linked with common variants for IBD (GWAS hits).

Open Targets interactome - Supplementary Information

The Molecular Interactions data aggregates and integrates interaction evidence reported in several resources to provide a systematic view on potentially relevant drug targets. Each of the integrated resources captures relationships of different nature including physical binary interactions, enzymatic reactions or functional relationships. The information here aims to capture not only the topology of the interaction network, but also the supporting experimental evidence reported on each of the databases.

In order to maximise coverage, the network contains all reported binary relationships between gene products (proteins and RNAs). Although the main focus are interactions between human molecules, the data also includes additional interactions between human gene products and molecules encoded in the genome of infectious pathogens - viruses and bacteria.

Data sources

IntAct

IntAct - http://www.ebi.ac.uk/intact - is a freely available, open source database for molecular interaction data. IntAct contains physical interactions derived from literature curation or direct user submissions.
Interactions are scored using the MI score. Benefiting from the PSI-MI controlled vocabulary, the Intact MI score provides a normalised (0 to 1) score that weights how recurrently an interaction has been reported, together with the confidence of the experimental techniques reported. Beware, a high scoring interaction can be due to high-confidence evidence, but also a social bias on studying certain proteins. Generally speaking, scores > 0.4 correspond to medium to high-confidence interactions, although some good-quality high-throughput interactions might still be scored below that threshold. More info on MI-score can be found in the Intact documentation.
Interactions are grouped by interaction detection method and interaction type. As a consequence, the same pair of interactors might be split into multiple entries if individual proteins are reported to have different biological roles.
For IntAct, please note:

- The network only contains human and selected pathogen data from IntAct
- The majority of interactions are not directional and not signed. However, there are a proportion of interactions where the biological role of the participants can be stated and directionality specified (e.g. enzymatic reactions)

Reactome

Reactome - https://reactome.org/ - is an open-source, open access, manually curated and peer-reviewed pathway database.
For Reactome, please note:

- Only human-human interactions are provided
- Interactions are directional and signed, with biological roles assigned to each participant if possible
- Protein interactions in Reactome are inferred from pathways and complexes based on Reactome internal method.

SIGNOR

SIGNOR, the SIGnaling Network Open Resource - https://signor.uniroma2.it/ - contains signaling information published in the scientific literature, which is manually-curated and stored in a structured format.
For SIGNOR, please note:

- SIGNOR only contains human data
- Interactions are directional and signed, with biological roles assigned to each participant
- The network pulls information from the SIGNOR relations file

STRING

STRING - https://string-db.org - contains functionally interacting proteins. While most interactions in the other resources capture different types of physical interaction between molecules, functional interactions do not necessarily interact physically. Both, direct (physical) and indirect (functional) associations are derived from computational predictions, from knowledge-transfer between organisms, or from interactions aggregated from other (primary) databases. STRING interactions provide an overall combined_score, as well as each of the pieces of information that compose this score. More information on STRING scoring can be found on their documentation page.

Computational pipeline and datasets

The multipartite network displayed in the Open Targets Platform is the result of post-processing the information stored in a Neo4j graph database (graphDB). The graphDB does not provide STRING information but contains ComplexPortal information on stable protein complexes as an additional data source. The information of the graphDB is then exported together with STRINGdb and mapped to the Open Targets Platform targets (Ensembl Gene IDs).

The resulting dataset as well as all intermediate files can be found in the Open Targets Platform Data Access section or the Intact FTP (ftp://ftp.ebi.ac.uk/pub/databases/intact/various/ot_graphdb/current/).
