# Supplementary material for: Network expansion of genetic associations defines a pleiotropy map of human cell biology
Source: Nat Genet. 2023 Feb 23;55(3):389–98. doi: 10.1038/s41588-023-01327-9 (PMC10011132; doi:10.1038/s41588-023-01327-9)
Supplement: Supplementary file 2 [file 41588_2023_1327_MOESM2_ESM.pdf]

nature portfolio

Corresponding author(s): Pedro Beltrao

Last updated by author(s): Aug 4, 2022

Reporting Summary

Nature Portfolio wishes to improve the reproducibility of the work that we publish. This form provides structure for consistency and transparency in reporting. For further information on Nature Portfolio policies, see our Editorial Policies and the Editorial Policy Checklist.

Statistics

For all statistical analyses, confirm that the following items are present in the figure legend, table legend, main text, or Methods section.

n/a	Confirmed	
☐	☒	The exact sample size (*n*) for each experimental group/condition, given as a discrete number and unit of measurement
☐	☒	A statement on whether measurements were taken from distinct samples or whether the same sample was measured repeatedly
☐	☒	The statistical test(s) used AND whether they are one- or two-sided<br>*Only common tests should be described solely by name; describe more complex techniques in the Methods section.*
☐	☒	A description of all covariates tested
☐	☒	A description of any assumptions or corrections, such as tests of normality and adjustment for multiple comparisons
☐	☒	A full description of the statistical parameters including central tendency (e.g. means) or other basic estimates (e.g. regression coefficient) AND variation (e.g. standard deviation) or associated estimates of uncertainty (e.g. confidence intervals)
☐	☒	For null hypothesis testing, the test statistic (e.g. *F*, *t*, *r*) with confidence intervals, effect sizes, degrees of freedom and *P* value noted<br>*Give P values as exact values whenever suitable.*
☒	☐	For Bayesian analysis, information on the choice of priors and Markov chain Monte Carlo settings
☐	☒	For hierarchical and complex designs, identification of the appropriate level for tests and full reporting of outcomes
☐	☒	Estimates of effect sizes (e.g. Cohen's *d*, Pearson's *r*), indicating how they were calculated

Our web collection on statistics for biologists contains articles on many of the points above.

Software and code

Policy information about availability of computer code

Data collection	No software was used for data collection
Data analysis	The network based method and all subsequent analysis was performed using R software (v 4.0.2) as described in material and methods, combined with the following packages: igraph (v 1.2.4.2, for Personalized Page Rank and walktrap clustering), pROC (v 1.16.2, for ROC curves and AUCs calculations when applicable), clusterprofiler (v4.2.2, for GOBP enrichment analysis in the description of the modules as well as GSEA test), pheatmap (v 1.0.12, for heatmap calculations when applicable), ggplot2 (v 3.3.2 for figure 5), vioplot (v 0.3.5 for violin plots) viridis (v 0.3.0) and RColorBrewer (v 1.1.2) both for color palette generation. The R functions used to perform the network expansion (Propagation using PPR and community detection to define gene modules) is publicly available in Github (https://github.com/ibarrioh/Network_expansion.git). Other scripts are available upon request to the corresponding author.

For manuscripts utilizing custom algorithms or software that are central to the research but not yet described in published literature, software must be made available to editors and reviewers. We strongly encourage code deposition in a community repository (e.g. GitHub). See the Nature Portfolio guidelines for submitting code & software for further information.

Data

Policy information about availability of data

All manuscripts must include a data availability statement. This statement should provide the following information, where applicable:

- Accession codes, unique identifiers, or web links for publicly available datasets
- A description of any restrictions on data availability
- For clinical datasets or third party data, please ensure that the statement adheres to our policy

All data generated or analysed during this study are included in this published article (and its supplementary information files). Publicly available repositories can be

access as follows:
OTAR interactome (ftp://ftp.ebi.ac.uk/pub/databases/intact/various/ot_graphdb/current), STRING v. 11.0 (https://string-db.org/), Open Targets Genetics portal (genetics.opentargets.org), Mouse KO phenotypes (IMPC, https://www.mousephenotype.org/), ClinVar (NCBI, https://www.ncbi.nlm.nih.gov/clinvar/), BioGRID Open Repository of CRISPR Screens (ORCS, v1.1.11, https://orcs.thebiogrid.org/), BiGRID v 4.4.202 for protein and genetic interactions (https://thebiogrid.org/), Human Protein Atlas (https://www.proteinatlas.org/), DISEASE database (diseases.jensenlab.org) and ChEMBL (https://www.ebi.ac.uk/chembl/)

Field-specific reporting

Please select the one below that is the best fit for your research. If you are not sure, read the appropriate sections before making your selection.

- [x] Life sciences
- [ ] Behavioural & social sciences
- [ ] Ecological, evolutionary & environmental sciences

For a reference copy of the document with all sections, see nature.com/documents/nr-reporting-summary-flat.pdf

Life sciences study design

All studies must disclose on these points even when the disclosure is negative.

Sample size	The sample size correspond to all the available data that was available for the analysis as specified in the figure or figure legends. No analysis was performed to determine the sample size prior to the analysis. Each statistical test performed takes into account the sample sizes appropriately.
Data exclusions	No data were excluded from the analyses.
Replication	No lab experiments were performed in this study. Replicability is performed in several analysis in the paper by comparing predictions against past true knowledge.
Randomization	No randomization was performed. In the network expansion analysis, permutations of the true positive set with random samples was used to determine potential biases in the placement of the true positives within the network.
Blinding	No lab experiments were performed and the data analysis was done on previously collected data and as such those analysing the results did not perform the data collection.

Reporting for specific materials, systems and methods

We require information from authors about some types of materials, experimental systems and methods used in many studies. Here, indicate whether each material, system or method listed is relevant to your study. If you are not sure if a list item applies to your research, read the appropriate section before selecting a response.

Materials & experimental systems

n/a	Involved in the study	
[x]	[ ]	Antibodies
[x]	[ ]	Eukaryotic cell lines
[x]	[ ]	Palaeontology and archaeology
[x]	[ ]	Animals and other organisms
[x]	[ ]	Human research participants
[x]	[ ]	Clinical data
[x]	[ ]	Dual use research of concern

Methods

n/a	Involved in the study	
[x]	[ ]	ChIP-seq
[x]	[ ]	Flow cytometry
[x]	[ ]	MRI-based neuroimaging
